# Psychological and weight history variables as predictors of short‐term weight and body fat mass loss

**DOI:** 10.1002/osp4.394

**Published:** 2019-12-02

**Authors:** Sharmin Akter, John A. Dawson, Chanaka N. Kahathuduwa, Shao‐Hua Chin, Martin Binks

**Affiliations:** ^1^ Department of Nutritional Sciences Texas Tech University Lubbock Texas; ^2^ Physician Assistant Program Midland Texas Tech University Health Sciences Center Lubbock Texas

**Keywords:** obesity, Power of Food Scale, Three Factor Eating Questionnaire, weight loss

## Abstract

**Objective:**

Identifying predictors of early weight loss may have value in predicting longer‐term success in weight loss programmes. This study examined if weight history variables (ie, weight cycling history [WCH], age of onset of obesity [AOO]), and preintervention Three‐Factor Eating Questionnaire (TFEQ) and Power of Food Scale (PFS) scores predicted weight loss (WL) and fat mass loss (FML) following a 3‐week calorie restriction intervention.

**Methods:**

Thirty‐two participants (19‐60 y; body mass index [BMI] 30‐39.9 kg/m^2^) participated in a 3‐week calorie restriction intervention (1120 kcal/d) as part of a larger clinical trial with 28 completers included in the current analyses. Preintervention WCH, AOO, TFEQ, and PFS subscale scores were collected, and WL and FML were measured. Multiple linear regression analyses were performed to predict WL and FML for relevant covariates in this study.

**Results:**

WCH, AOO, preintervention TFEQ subscale scores, and PFS subscale scores did not predict WL (all *P*s > .08) or FML (*P*s > .06) except, PFS‐food tasted scores significantly predicted WL (*r* = −0.40, *P* = .03).

**Conclusion:**

Although these variables were not robust predictors, results for at least the PFS suggest there may be value in further exploring this measure using larger sample sizes.

## 1 INTRODUCTION

There is increasing evidence that early weight loss (as early as 1‐3 wk) is predictive of later weight loss/treatment response^1^ and may also predict longer‐term outcomes.^2^, ^3^, ^4^ While some attempts have been made to characterize predictors of treatment response, these typically look at baseline predictors of distal outcomes (several months) with little attention paid to the perhaps crucial “very early” response to treatment. Typically, the majority of people undergoing dietary interventions do well in the initial few weeks with increasing heterogeneity of success among individuals with increasing time in treatment. The very early stage of weight loss (initial 3 wk) represents an as yet understudied gap in understanding of treatment response. Some common psychosocial, behavioural, and physiological factors within the context of very early treatment response may provide predictors that are informative in understanding contributors to early success or lack thereof and allow us to identify potential targets for adjusting treatment early in those at risk for nonresponse. These predictors may be different for dietary interventions utilizing typical foods eaten vs total meal replacement (TMR) programs based on the known differences of these diet types in terms of both treatment outcomes and psychological response to food cues.^5^, ^6^

Factors related to weight history such as weight cycling (repeated weight loss and regain) and age of onset of obesity have received only moderate attention in the obesity literature to date with somewhat equivocal findings. Weight cycling in individuals with obesity and those who do not yet have obesity has been associated with a higher risk of developing or exacerbating obesity and also with increased abdominal and visceral fat accumulation.^7^, ^8^, ^9^, ^10^ However, in terms of predicting successful weight loss, the association of weight cycling and body weight change is inconclusive.^11^, ^12^, ^13^ There is some suggestion that history of weight cycling may act as both an impedance to weight loss success in those undergoing treatment and may also be useful as a predictor of future weight gain.^11^, ^14^, ^15^

Early age of onset of obesity has been associated with an increased incidence of adult obesity, increased overall adiposity, as well as the number of adipose cells in the body.^16^, ^17^, ^18^ One study suggests that children and adolescents with obesity are five times more likely to develop obesity in adult age than those who did not have obesity at an early age.^19^ Some limited evidence suggests that age of onset may not influence success during weight loss treatment,^20^, ^21^ while at least one study reported that during 1 to 2 years follow up after bariatric surgery, early age of onset was associated with less weight loss.^22^

Psychological influences on eating behaviour have long been of interest in relation to predicting weight loss outcomes. Numerous self‐report measures have been developed in an attempt to characterize the relationship with food in people with obesity and subsequently provide quantifiable measures of potential psychological predictors. The Three Factor Eating Questionnaire (TFEQ)^23^ is a widely used and longstanding instrument of this kind. In its original 51‐item version, it attempts to quantify eating behaviours along three distinct general psychological dimensions: (a) cognitive restraint, exerting cognitive control to restrict food intake; (b) disinhibition, the experience of impulsive/uncontrolled eating; and (c) susceptibility to hunger, which characterizes the likelihood of eating while in a hunger state. The scale identifies individual patterns of eating behaviour that may lead to overconsumption of foods based on specific psychological states/dysregulation. Higher scores on the TFEQ are associated with higher body mass index (BMI).^24^, ^25^ Several studies have found that TFEQ scores can predict subsequent success with weight loss.^26^, ^27^

The Power of Food Scale (PFS) measures the psychological impact of living in food‐abundant environments. Specifically, the PFS quantifies appetite for palatable foods in the context of availability, proximity, and personal experience of a food (ie, food available, food present, and food tasted).^28^ The measurement of hedonically based motivations to eat in environmental context has utility in predicting long‐term weight control.^28^, ^29^ Understanding how these well‐validated psychological measures are related to very early treatment response will provide valuable insights into identifying early responders to dietary intervention.

The purpose of this study was to determine the predictive value of preintervention weight history and psychological predictor variables such as history of weight cycling, age of onset of obesity, and current relationship with food as measured by TFEQ subscales (dietary restraint, disinhibition, and susceptibility to hunger), and PFS scores (food available, food presented, food tasted) in predicting the short‐term (initial) treatment response (ie, body weight loss [BWL] and fat mass loss [FML]) in relation to two types of dietary intervention (TMR vs typical diet [TD]).

## 2 METHODS

### 2.1 Participants

Thirty‐two adults (M = 31.68 ± 12.98 y) with obesity (BMI 30‐39.9 kg/m^2^) who enrolled in a larger weight loss intervention/functional magnetic imaging study conducted at the Behavioural Medicine and Translational Research (BMTR) Lab at Texas Tech University were eligible for inclusion in this study. Data from 28 completers were analysed. Details of the original study are reported elsewhere.^5^ Potential participants were contacted via telephone for eligibility screening. The exclusion criteria for the original study were as follows: motor, visual, and auditory impairment; contraindication for magnetic resonance imaging; women with irregular menstrual cycle, pregnant or nursing; medical, psychological, and neurological contradictions (hypertension, diabetes mellitus, severe depression, eating disorder, etc); current medication that may affect study outcome variables; history of bariatric surgery or recent weight loss treatment (3 mo); current smokers; and unwillingness to provide informed consent.

### 2.2 Design overview

Participants who met all eligibility criteria were enrolled in the original study. Participants attended visit 1 for baseline (preintervention) measurement: (a) self‐report assessment measures (BMTR Lab Weight History form (included age of onset, weight cycling history)), (b) measured height via stadiometer (HR‐200 TANITA wall‐mounted height rod, TANITA Corporation of America Inc, Illinois), and (c) measured preintervention body weight, body fat mass, and BMI via bioelectrical impedance analysis (TANITA Corporation of America Inc, Illinois, BC‐418 segmental body composition analyser). If all inclusion criteria were met, informed consent was obtained and participants attended visit 2 where they completed the preintervention self‐report measures related to the relationship with food (ie, TFEQ and PFS) followed by the fMRI scan (note fMRI data were not included in the current study) after which they were randomly assigned to either TMR with Optifast 800 shakes or TD groups. Participants attended two check‐in visits between assessment visits. After completion of the 3‐week dietary intervention, (visit 5) body weight, body fat mass was measured.

### 2.3 Dietary intervention

Immediately following the initial fMRI scanning conducted in visit 2, all the participants were randomly assigned to either TMR or TD group.

### 2.4 TMR group

Every participant assigned to the TMR group was instructed in the appropriate use of the meal replacement product. Optifast 800 TMR shakes (Nestlé HealthCare Nutrition Inc, New Jersey) were provided in a quantity that would last for a 1‐week period with the prescribed energy intake of 1120 kcal/d. Every participant was instructed to abstain from all items of food for this 3‐week intervention period, except the meal replacement product and water/noncaloric beverages. Participants were requested to return to the BMTR lab after completion of each week consecutively for 2 weeks to receive next week's product and to be weighed, have blood pressure measured, and speak with an investigator to review any issues related to diet adherence.

### 2.5 TD group

Participants assigned to the TD group were instructed to use portion control to maintain their daily calorie intake at 1120 kcal/d but not make significant changes in typical foods eaten. Participants were directed to return to the BMTR lab at every 1‐week interval over the following 2 weeks to measure their weight and blood pressure and to review any issues related to diet adherence.

### 2.6 Self‐report measures

The BMTR Demographic, Health, and Weight History Form is a self‐report measure used to collect basic demographic, medical, and psychosocial information. This study used the following data from this form: (a) history of weight cycling; participants were asked “How many times have you lost 10 lbs. or more when you weren't sick and then gained it back (excluding childbirth)?” Response options were as follows: (1) never; (2) once or twice; (3) three or four times; (4) five times or more; (b) age of onset of overweight or obesity; participants were asked, “At what age did you first consider yourself overweight?” Response options were as follows: (1) never; (2) childhood (0‐12); (3) adolescence (13‐17); (4) young adult (18‐25); (5) adult (26‐49); (6) late adult (50+).

The TFEQ is a validated self‐report questionnaire that measures three factors associated with ingestive behaviour: dietary restraint, disinhibition, and susceptibility to hunger.^23^ The dietary restraint scale refers to an intention to control food intake in an effort to control weight and consists of 21 items. Disinhibition measures the loss of control in relation to ingestive behaviour and contains 16 questions. Susceptibility to hunger measures an individual's perception of intent towards food intake relative to hunger. It has 14 questions. The scale has moderate internal consistency (Cronbach α.73‐.90) and high test‐retest reliability (0.8‐0.91).^23^, ^30^ The measurement consists of two parts. The first part consists of 36 true‐false questions scoring either a 0 or 1 point depending on the response. In the second part, 15 multiple choice questions with four/five options marked either as 1 to 4 or 1 to 5 are asked. Based on specific scoring instructions, each response is coded as either 0 or 1.

The PFS is a validated self‐report measure that assesses the psychological impact of living in a food abundant environment.^28^ PFS items were designed to reflect three levels of food proximity: (1) food available but not physically present, (2) food present but not tasted, and (3) food tasted but not consumed. The PFS consists of 15 questions and response options are as follows: (1) I don't agree, (2) I agree a little, (3) I agree somewhat, (4) I agree quite a bit, (5) I strongly agree. All the scales are scored individually according to published guidelines, and these scales are summed to produce the total score. From recently available data, the test‐retest reliability was adequate (0.71) with good internal consistency (Cronbach *α*.91).^28^

### 2.7 Statistical analysis

Data were examined using the R statistical software package (version 3.4.3).^31^ All continuous variables (eg, TFEQ score, PFS score, weight, and fat mass) were examined for normality and outliers and for compliance with the assumptions of linear regression. Descriptive statistics of all the measured variables were calculated and reported as mean and standard deviation (M ± SD). Chi‐square tests of homogeneity were used to interrogate categorical variables for potential differences by sex or diet grouping.

Multiple linear regression analyses were performed to predict postintervention vs preintervention changes in body weight and fat mass using preintervention TFEQ subscales, preintervention PFS score, age of onset of obesity and weight cycling as predictors (separately) adjusting for age, sex, diet group, and preintervention body weight, fat mass, and BMI. Stepwise model selection was used to report the best model as chosen by Akaike information criterion (AIC), where a lower AIC value denotes a better model.

Univariate linear models were used in order to assess various bivariate associations between continuous and categorical measurements. Additionally, simple linear regression models were used in order to estimate point estimates and confidence intervals for various bivariate correlations among the continuous measurements. Nominal significance using an alpha threshold of.05 is used throughout.

### 2.8 Ethics

This study was approved by the Texas Tech University, Human Subjects, Institutional Review Board (TTU IRB #505380). The original study is registered in http://Clinicaltrials.gov (NCT02637271). All procedures were carried out in accordance with the Declaration of Helsinki, 2000.^32^ Upon meeting the eligibility criteria, a written informed consent was obtained from all participants.

## 3 RESULTS

**Figure 1:**
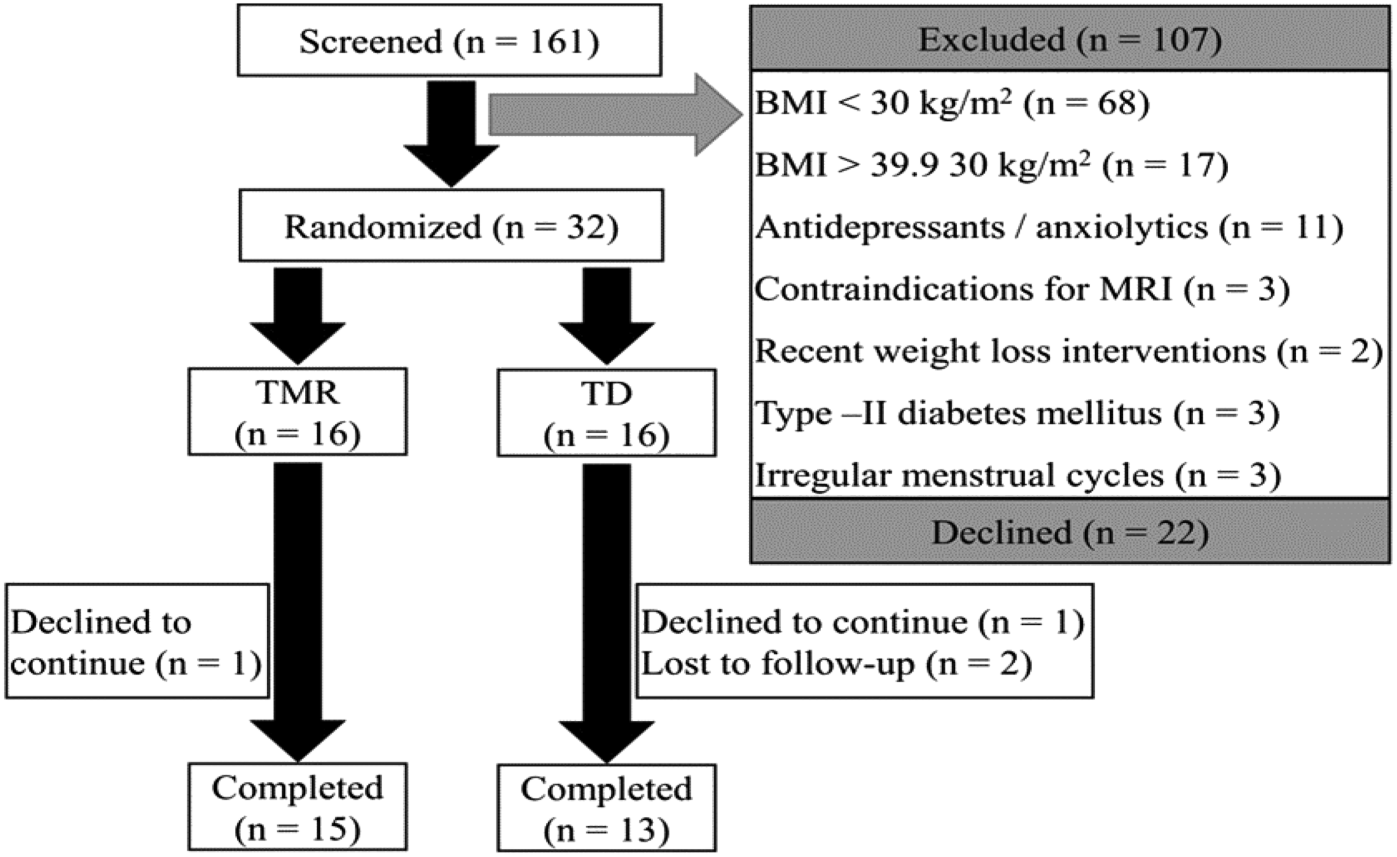
Eligibility and randomization of study participants and completion of data collection—CONSORT diagram

Fifteen participants in the TMR group and thirteen participants in the TD group completed the larger study (see Figure 1: CONSORT diagram) and were included in the analyses. The two groups did not differ by age, sex, weight, fat mass, height, and BMI. The preintervention characteristics of this study are presented in Table 1, and weight history data with frequency are presented in Table 2.

**Table 1:** Preintervention characteristics of study participants (n = 28)

Measurement	Mean ± SD	95% CI
Weight, kg	98.94 ± 16.43	[92.57, 105.31]
Height, cm	167.80 ± 11.04	[163.52, 172.08]
BMI, kg/m^2^	34.99 ± 3.22	[33.74, 36.24]
Fat mass, kg	39.08 ± 10.50	[35.00, 43.15]
Age	31.68 ± 12.98	[26.64, 36.71]
TFEQ	24.21 ± 6.97	[21.51, 26.92]
TFEQ‐R	9.14 ± 4.52	[7.39, 10.90]
TFEQ‐D	8.25 ± 3.92	[6.73, 9.77]
TFEQ‐S	6.82 ± 3.31	[5.54, 8.11]
PFS	3.02 ± 0.84	[2.70, 3.35]
PFS‐FA	2.64 ± 0.88	[2.30, 2.98]
PFS‐FP	3.49 ± 1.20	[3.02, 3.96]
PFS‐FT	3.11 ± 0.78	[2.80, 3.41]

Abbreviations: BMI, body mass index; PFS, Power of Food Scale; PFS‐FA, PFS‐food available; PFS‐FP, PFS‐food present; PFS‐FT, PFS‐food tasted; TFEQ, Three Factor Eating Questionnaire; TFEQ‐D, TFEQ‐Disinhibition Scale; TFEQ‐R, TFEQ‐Restraint Scale; TFEQ‐S, TFEQ‐Susceptibility to Hunger Scale.

**Table 2:** Preintervention weight history data

Weight Cycling	Frequency	Age of Onset of Obesity	Frequency
Never	4	Never	1
Once or twice	15	Childhood	8
Three or four times	6	Adolescence	4
Five times or more	3	Young adult	8
		Adult	6
		Late adult	1

After randomly assigning participants into TMR and TD groups, history of weight cycling and age of onset of obesity showed no significant difference between sex (male and female) and diet group (TMR and TD) (Table 3). Also, there was no difference between male and female distribution within the diet groups (Table 4). Cross‐tabulations were analysed using chi‐square tests of independence. The 3‐week calorie‐restricted diet resulted in a significant reduction in body weight and body fat mass (Tables 5 and 6).

**Table 3:** Weight history data within sex (male and female) and group (TMR and TD)

Weight Cycling	Frequency		*P* Value	Age of Onset of Obesity	Frequency		*P* Value
	Male	Female			Male	Female	
Never	2	2	.95	Never	1	0	.75
Once or twice	6	9		Childhood	3	5	
Three or four times	3	3		Adolescence	2	2	
Five times or more	1	2		Young adult	4	4	
				Adult	3	4	
				Late adult	0	1	
	TMR	TD	.59		TMR	TD	.62
Never	3	1		Never	0	1	
Once or twice	8	7		Childhood	5	3	
Three or four times	2	4		Adolescence	2	2	
Five times or more	2	1		Young adult	5	3	
				Adult	2	4	
				Late adult	1	0	

Abbreviations: TD, typical diet; TMR, total meal replacement.

**Table 4:** Male and female distribution within diet groups (TMR and TD)

	Total Meal Replacement (TMR)	Typical Diet (TD)	*P* value
Male	8	4	.41
Female	7	9	

**Table 5:** Postintervention anthropometric measurement

Measurement	Mean ± SD	95% CI
Weight, kg	95.23 ± 15.90	[89.07, 101.40]
Fat mass, kg	37.14 ± 11.28	[32.77, 41.52]
BMI, kg/m^2^	33.89 ± 3.49	[32.53, 35.24]

**Table 6:** Postintervention changes in body weight and fat mass

Measurement	Mean ± SD	95% CI	*t* Value	*P* value
Weight loss	3.71 ± 2.53	[2.73, 4.69]	7.75	2.46e−8
Fat mass loss	1.93 ± 2.03	[1.14, 2.72]	5.03	2.81e−5

*Note*. Weight loss and fat mass loss were analysed with Welch‐Satterthwaite *t* test.

### 3.1 Association of weight history variables and BWL and FML

Data from history of weight cycling and from age of onset of obesity were analysed to determine correlations with BWL and FML (Tables 7 and 8). The analyses showed that age of onset of obesity and history of weight cycling were not associated with BWL and FML.

**Table 7:** Association between history of weight cycling and BWL and FML

Variable	BWL		FML	
	Estimate	Std. Error	Estimate	Std. Error
Intercept	4.98	1.24	2.93	1.04
Never	‐‐	‐‐	‐‐	‐‐
Once or twice	−1.09	1.39	−1.19	1.17
Three or four times	−2.94	1.60	−1.58	1.34
Five times or more	−0.51	1.89	−0.24	1.58
	*F* statistic = 1.38; *R* ^2^ = .14; *P* = .27		*F* statistic = 0.64; *R* ^2^ = .07; *P* = .60	

Abbreviations: BWL, body weight loss; FML, fat mass loss.

**Table 8:** Association between age of onset of obesity and BWL and FML

Age of Onset	BWL		FML	
	Estimate	Std. Error	Estimate	Std. Error
Intercept	3.76	1.98	8.30	2.47
Never	‐‐	‐‐	‐‐	‐‐
Childhood	−1.65	2.10	−5.25	2.62
Adolescence	−3.70	2.21	−4.50	2.76
Young adult	−0.94	2.10	−3.73	2.62
Adult	−2.12	2.14	−5.33	2.67
Late adult	−2.86	2.80	−6.80	3.49
	*F* statistic = 1.27; *R* ^2^ = .22; *P* = .31		*F* statistic = 1.30; *R* ^2^ = .23; *P* = .30	

Abbreviations: BWL, body weight loss; FML, fat mass loss.

When using multiple linear regression and stepwise model selection to allow for the inclusion of other relevant study covariates in addition to history of weight cycling, the best model (*R* ^2^ = .65; *P* value = 1.64e−3) for predicting BWL contained diet group (*P* = 1.03e−3), preintervention weight (*P* = 7.49e−4), and BMI (*P* = 1.92e−3). Weight cycling was not a significant predictor after stepwise model selection.

Likewise, when using multiple linear regression and stepwise model selection to allow for the inclusion of other relevant study covariates in addition to age of onset, the best model (*R* ^2^ = .71, *P* value = 2.26e−3) for predicting BWL contained diet group (*P* = 4.27e−4), preintervention weight (*P* = .03), and fat mass (*P* = .04); however, age of onset of obesity similarly did not survive model selection. Similarly, neither age of onset nor weight cycling history were significant predictors of FML when allowing for the inclusion of other covariates.

### 3.2 Association of TFEQ and PFS subscales with BWL and FML

Linear modelling showed that the correlations between preintervention TFEQ subscales and PFS subscales with body weight and fat mass loss were not significant, except PFS‐Food Tasted score was negatively correlated with BWL (*P* = .03) (Tables 9 and 10).

**Table 9:** Linear correlation coefficients between preintervention TFEQ subscales and BWL and FML

	Model	Variable	Estimate	Std. Error	*t* Value	*P* value	95% CI
BWL	1	Intercept	4.55	1.78	2.55	.02	
		TFEQ	−0.03	0.07	−0.49	.63	
		*r* = −0.10; *R* ^2^ = 9.23e−3; *P* value = .63					[−0.45, 0.29]
	2	Intercept	4.18	1.11	3.76	8.72 e−4	
		TFEQ‐R	−0.05	0.11	−0.47	.64	
		*r* = −0.09; *R* ^2^ = 8.47e−3; *P* value = .64					[−0.45, 0.29]
	3	Intercept	4.11	1.15	3.58	1e−3	
		TFEQ‐D	−0.05	0.13	−0.39	.70	
		*r* = −0.08; *R* ^2^ = 5.77e−3; *P* value = .70					[−0.44, 0.31]
	4	Intercept	3.64	1.13	3.21	3.50e−3	
		TFEQ‐S	0.01	0.15	0.07	.95	
		*r* = 0.01; *R* ^2^ = 1.80e−4; *P* value = .95					[−0.36, 0.38]
FML	1	Intercept	3.41	1.41	2.42	.02	
		TFEQ	−0.06	0.06	−1.09	.29	
		*r* = −0.21; *R* ^2^ = .04; *P* value = .29					[−0.54, 0.18]
	2	Intercept	1.43	0.89	1.61	.12	
		TFEQ‐R	0.06	0.09	0.63	.54	
		*r* = 0.12; *R* ^2^ = .02; *P* value = .53					[−0.26, 0.47]
	3	Intercept	3.12	0.89	3.50	1.69e−3	
		TFEQ‐D	−0.14	0.10	−1.47	.15	
		*r* = −0.28; *R* ^2^ = .08; *P* value = .15					[−0.59, 0.11]
	4	Intercept	3.10	0.87	3.55	1.50e−3	
		TFEQ‐S	−0.17	0.12	−1.48	.15	
		*r* = −0.28; *R* ^2^ = .08; *P* value = .15					[−0.59, 0.10]

Abbreviations: BWL, body weight loss; FML, fat mass loss; TFEQ, Three Factor Eating Questionnaire; TFEQ‐D, TFEQ‐Disinhibition Scale; TFEQ‐R, TFEQ‐Restraint Scale; TFEQ‐S, TFEQ‐Susceptibility to Hunger Scale.

**Table 10:** Linear correlation coefficients between preintervention PFS subscales and BWL and FML

	Model	Variable	Estimate	Std. Error	*t* Value	*P* Value	95% CI
BWL	1	Intercept	6.95	1.73	4.02	4.43e−4	
		PFS	‐1.07	0.55	−1.94	.06	
		*r* = −0.36; *R* ^2^ = .13; *P* value = .06					[−0.64, 0.02]
	2	Intercept	5.64	1.52	3.72	9.63e−4	
		PFS‐FA	−0.73	0.55	−1.34	.19	
		*r* = −0.26; *R* ^2^ = .06; *P* value = .19					[−0.57, 0.13]
	3	Intercept	6.12	1.44	4.26	2.37e−4	
		PFS‐FP	−0.69	0.39	−1.77	.09	
		*r* = −0.33; *R* ^2^ = .11; *P* value = .09					[−0.63, 0.05]
	4	Intercept	7.76	1.86	4.18	2.94e−4	
		PFS‐FT	−1.31	0.58	−2.25	.03	
		*r* = −0.40; *R* ^2^ = .16; *P* value = .03					[−0.67, 0.04]
FML	1	Intercept	4.45	1.40	3.19	3.71e−3	
		PFS	−0.83	0.45	−1.87	.07	
		*r* = −0.34; *R* ^2^ = .12; *P* value = .07					[−0.64, 0.03]
	2	Intercept	3.86	1.20	3.23	3.34e−3	
		PFS‐FA	−0.73	0.43	−1.70	.10	
		*r* = −0.32; *R* ^2^ = .10; *P* value = .10					[−0.62, 0.06]
	3	Intercept	3.49	1.18	2.96	6.47e−3	
		PFS‐FP	−0.45	0.32	−1.40	.17	
		*r* = −0.26; *R* ^2^ = .07; *P* value = .17					[−0.58, 0.12]
	4	Intercept	4.82	1.52	3.16	3.97e−3	
		PFS‐FT	−0.93	0.47	−1.95	.06	
		*r* = −0.36; *R* ^2^ = .13; *P* value = .06					[−0.64, 0.02]

Abbreviations: BWL, body weight loss; FML, fat mass loss; PFS, Power of Food Scale; PFS‐FA, PFS‐food available; PFS‐FP, PFS‐food present; PFS‐FT, PFS‐food tasted.

Multiple linear regression analyses were conducted to predict BWL and FML by preintervention TFEQ subscales while controlling for relevant covariates in this study (ie, diet group, age, gender, preintervention weight, fat mass, and BMI). After stepwise model selection, the best model based on AIC was the interaction model with TFEQ subscale and diet group predicting BWL (*R* ^2^ = .73, *P* value = 1.30e−3). In this model, high score in TFEQ‐Disinhibition was correlated with higher weight loss (*P* value = 1.07e−3), high score in TFEQ‐Restraint, and TFEQ‐Hunger were correlated with lower weight loss (*P* value: 2.96e−3, 1.38e−3, respectively) (Table 11). Also, preintervention weight (*P* value = .04), interaction with TMR diet group, and preintervention TFEQ‐Disinhibition (*P* value = 3.15e−3), TFEQ‐Restraint (*P* value = 6.35e−3), and TFEQ‐Hunger (*P* value = 3.00e−3) were significant predictors in this model. The models for TFEQ subscales and FML were not significant.

**Table 11:** Multiple linear regression model for TFEQ and BWL

	Coefficients	Estimate	Std. Error	Test Statistic	*P* value
BWL	Intercept	3.51	3.03	1.34	.26
	TFEQ‐D	1.06	0.27	15.16	1.07e−3
	TFEQ‐R	−0.50	0.15	11.79	2.96e−3
	TFEQ‐S	−1.37	0.36	14.27	1.38e−3
	Diet group (TMR)	−1.99	2.48	0.64	.43
	Preintervention weight	0.06	0.03	5.02	.04
	Preintervention fat mass	−0.06	0.04	2.58	.13
	TFEQ‐D and TMR	−1.15	0.34	11.59	3.15e−3
	TFEQ‐R and TMR	0.53	0.17	9.53	6.35e−3
	TFEQ‐S and TMR	1.45	0.43	11.14	3.67e−3
*F* statistic = 5.31; multiple *R* ^2^ = .73; omnibus *P* value = 1.30e−3					

Abbreviations: TFEQ, Three Factor Eating Questionnaire; TFEQ‐D, TFEQ‐Disinhibition Scale; TFEQ‐R, TFEQ‐Restraint Scale; TFEQ‐S, TFEQ‐Susceptibility to Hunger Scale; TMR, total meal replacement.

Multiple linear regression analyses were conducted to predict BWL and FML with PFS subscales while controlling for relevant covariates in this study. The best model for predicting BWL with PFS subscales showed a significant 70% variance (*R* ^2^ = .70, *P* value = 1.07e−4). However, the interaction between PFS subscales and diet group was not significant.

Furthermore, the best model for predicting FML by PFS subscales while controlling for covariates for this study was the interaction model of PFS subscale and diet group (*R* ^2^ = .49, *P* value = .04), with significant partial effects of TMR diet group (*P* = 3.57e−3), preintervention fat mass (*P* = .03), and the interaction between PFS‐Food Available score and TMR diet group (*P* = 5.48e−3) to the full model. (Table 12).

**Table 12:** Multiple linear regression model for PFS and FML

	Coefficients	Estimate	Std. Error	Test Statistic	*P* value
FML	Intercept	−3.19	3.17	1.01	.37
	PFS‐FA	0.67	0.54	1.54	.23
	Diet group (TMR)	7.25	2.20	10.90	3.57e−3
	Age	0.05	0.03	3.29	.09
	Preintervention weight	0.08	0.04	3.78	.07
	Preintervention fat mass	−0.15	0.06	5.55	.03
	Gender	−2.20	1.24	3.25	.09
	PFS‐FA and TMR	−2.41	0.77	9.69	5.48e−3
*F* statistic = 2.76; Multiple *R* ^2^ = .49; Omnibus *P* value = .04					

Abbreviations: PFS‐FA, PFS‐Food Available; TMR, Total Meal Replacement (TMR).

## 4 DISCUSSION

This study considered whether weight history and psychological relationship with food variables were associated with BWL and FML in a sample of adults with class I and II obesity undergoing a 3‐week iso‐caloric TMR‐ and TD‐based weight loss intervention. History of weight cycling and age of onset were not significantly associated with initial, short‐term BWL and FML in univariate models. For all the categories in the history of weight cycling variables (never, once or twice, etc), there were no significant predictors noted for BWL. Similar results were found from the selected model of age of onset to predict BWL. This result does not support the hypothesis that BWL and FML would be predicted by weight history variables. Based on the literature, history of weight cycling and early age of onset may act as an impedance to weight loss^14^, ^33^; however, a few studies with adult women reported that history of weight cycling^34^ and age of onset^21^ has no effect on weight loss. The scarcity of research in this area limits the ability to explain why weight cycling and age of onset did not predict BWL and FML in this study beyond what has already been reported in the literature. One possible explanation may be found in the primary aim of the study, which was limited to understanding “early” weight loss. The variability in the literature may be indicative of a wide range of treatment durations. Thus, while over the longer term these types of history variables may become relevant during the course of longer treatments, they do not appear to influence early response.

Overall, the hypotheses that TFEQ and PFS subscales would negatively predict BWL and FML were not supported; this was somewhat unexpected in the context of the literature..^27^, ^35^, ^36^ There are two primary differences that should be noted: (a) These studies typically included larger sample sizes, and (b) the referenced studies typically included a wider BMI range (this study sample is limited to class I and II obesity). In this study, the combination of having a small sample size and narrow range of BMI might have resulted in reduced variability in TFEQ scores since people with high BMI are more likely to report high scores on TFEQ subscales creating homogeneity that would be less likely if the full range of typical scores were included. In addition, a smaller sample limits the number of individual data points also resulting in reduced variability in the sample. Together, this suggests the possibility that with larger sample size and a wider BMI range, the anticipated outcomes more in line with the literature may be seen. Alternatively, similar to what was stated above, what this study may be seeing is a unique relationship of these factors to the brief initial phases of weight loss (ie, 3 wk). While these simply represent plausible conjecture, nonetheless these findings will inform future directions in this regard.

Very limited evidence is available on the predictive power of PFS scores on BWL and FML.^37^, ^38^ A higher PFS score is associated with higher BMI, high food craving, and also with eating behaviour–related disorders such as binge eating. This study did not find any significant association between PFS score and BWL and FML except one (nominally) significant subscale finding. Specifically, an increased score in the PFS‐food tasted (PFS‐FT) scale was negatively associated with BWL. This is interesting, but interpretive caution is warranted. One plausible explanation is found in the fact that the questions from the PFS‐FT scale reflect a general tendency towards hedonic hunger and preoccupation with food regardless of homeostatic hunger. This is related to determining individual differences in the motivation to ingest highly palatable food in the food‐abundant environment.^28^ Preintervention scores on the PFS‐FT reflects participants' tendency towards overeating before starting the weight loss treatment. Thus, according to Didie et al, a high score in this scale may predict the likelihood of participants having high motivation to ingest more food that subsequently may cause lower intervention compliance and thus lower weight loss.^39^

In the multiple linear models predicting BWL with TFEQ subscales, an interaction with diet group was able to account for 73% of the variance in BWL. Predictor variables, such as TFEQ‐Disinhibition, preintervention weight, interaction within TFEQ‐Restraint with TMR diet group, and interaction with TFEQ‐Hunger and TMR diet group had a significant positive effect on this model. Variables such as TFEQ‐Restraint, TFEQ‐Hunger, and the interaction between TFEQ‐Disinhibition had a significant negative effect on predicting BWL. More importantly, the coefficients from the diet group by subscale interactions are essentially inverses of the subscale main effects. Another interesting finding is the interaction model of PFS and diet group when predicting FML.

Along with TMR diet group and preintervention fat mass, the interaction between PFS‐food available and TMR group had significant influence in predicting FML. Together, these findings can be interpreted in the following way. Contrary to what one might predict based on the general literature (ie, people scoring high on TFEQ are susceptible to the food environment), individuals in TMR diet group appear to not be susceptible to maladaptive eating behaviour (as measured by TFEQ and PFS). This is demonstrated by a lack of negative impact on weight loss or fat loss in the group on the TMR diet. An explanation for this lies in the nature of the TMR diet. TMR is thought to act in part via a narrowing of the stimulus environment as it relates to food cues. In other words, participants on TMR diet are thought to be deliberately abstaining from food‐related cues such as sight or smell of food, shopping for food, eating regular food, and also they selectively do not attend to advertisements for food and often avoid locations where food is present. If this indeed is the case (it is widely and consistently reported anecdotally in clinical settings), by avoiding these food cues, and by extension the presence of food, the vulnerability measured by the TFEQ and PFS is rendered moot. However, due to the low sample size in this study, low variability in observed TFEQ and PFS subscale values, and the fact that these interactions have a relatively small magnitude, caution must be exercised, and further research is needed to confirm whether this interaction‐based relationship is real or simply an artefact of this particular sample.

This study has several strengths: first, using two of the most commonly used and well‐validated questionnaires for measuring eating behaviours: TFEQ and PFS. Second, this study looked at two distinctly different iso‐caloric (1120 kcal/d) intervention types, TMR compared with TD, each with unique impacts on dietary behaviour and weight loss, thus allowing us to differentiate the ingestive behaviour of being in a food‐type restricted and equivalently calorie restricted environment. Another strength was that this study controlled for age, sex, diet group, gender, and preintervention weight, fat mass, and BMI in the exploratory analyses. All of these variables may significantly contribute BWL and FML, so their inclusion helps untangle their potential influences on the outcomes of primary interest. Finally, the research design, data analysis approach, and statistical reporting improves substantially on several deficiencies in the existing literature, which will provide a strong foundation from which to construct future studies examining this novel approach to the topics.

This study has some limitations. The lack of significant associations with simple linear modelling between predictor variables and BWL and FML versus a significant association between predictor variables and BWL and FML with multiple linear modelling suggest that this study may be lacking enough power (due to relatively low sample size or narrow ranges of covariate values). For example, using a narrow BMI range may have limited the range of scores on all the predictor variables since each of the variables is independently associated with BMI. Thus, having a high BMI score will more likely lead to reporting a high score on this survey measurement subscales, causing less overall variability in the total scores of the TFEQ and PFS instruments. Moreover, while this study observed a significant loss of weight and fat mass during the 3‐week intervention, these losses are only over a short time frame and may solely be specific to a population with high BMI. Thus, a larger sample size with wider BMI and other covariates ranges, as well as a longer intervention period, is prudent.

## 5 CONCLUSION

In conclusion, weight history variables (ie, weight cycling history and age of onset of overweight or obesity) did not predict BWL and FML following a 3‐week calorie restriction intervention. Scores of TFEQ subscales (ie, dietary restraint, disinhibition, and susceptibility to hunger) did not predict BWL and FML. Scores of PFS (ie, food available, food present, and food tasted) were not a robust predictor of BWL and FML. The results as a whole suggest that being in the TMR group has inverse effects on TFEQ subscales when predicting BWL and on PFS scores when predicting FML. A possible explanation may be that the TMR diet is thought to act through a narrowing of the food stimulus environment. In this context, this result makes intuitive sense: A person on TMR is not being exposed to the food environment; they are not shopping for food or cooking, and they often report consciously trying to avoid food commercials, restaurants, or other situations heavy with food cues. Thus, while they may be more susceptible in the food environment, the motivation towards ingesting food is negated by the dearth of cues. Furthermore, this was not seen in the TD group where the exposure in the food environment remains relatively unchanged, lending further support for this potential explanation of these findings.

## CLINICAL TRIALS REGISTRY NUMBER

NCT02637271; the protocol is available at https://clinicaltrials.gov/ct2/show/NCT02637271.

## AUTHOR CONTRIBUTIONS

All authors contributed substantively to the intellectual development of the manuscript. The corresponding author, Martin Binks, PhD, is principle investigator and oversaw all aspects of the conceptualization, design, and conduct of the study including but not limited to the following: data collection, management, analyses, and interpretation and preparation of subsequent study reports and manuscripts. Sharmin Akter, MS, was primary author and conducted statistical analyses. Chanaka Kahathuduwa, PhD, and Shao‐Hua Chin, PhD, conducted the data collection and contributed to the writing of the manuscript. John A. Dawson, PhD, contributed substantially to the design and conduct of the statistical analyses and writing of the manuscript.

## CONFLICT OF INTEREST STATEMENT

Corresponding author, Martin Binks, PhD, reports that the original data collection for this study was funded by Nestlé Health Science Inc. The funding agency was not involved in decisions related to the publication of outcomes. Dr Binks has also received honoraria in the past for presenting educational content on behalf of Nestlé Health Science Inc. Dr Binks further attests that this manuscript has neither been submitted for consideration nor been published elsewhere and represents an original work. The authors have no other potential conflicts of interest to declare.

## FUNDING SOURCE

The study was funded by Nestlé Health Science Inc.
